# Polygenic expression of teratozoospermia and normal fertility in B10.MOL‐TEN1 mouse strain

**DOI:** 10.1111/cga.12102

**Published:** 2015-04-16

**Authors:** Keitaro Hirawatari, Naoto Hanzawa, Maki Kuwahara, Hiroaki Aoyama, Ikuo Miura, Shigeharu Wakana, Hideo Gotoh

**Affiliations:** ^1^ Animal Genome Research Unit, Agrogenomics Research Center National Institute of Agrobiological Sciences Tsukuba Ibaraki Japan; ^2^ Technology and Development Team for Mouse Phenotype Analysis RIKEN BioResource Center Tsukuba Ibaraki Japan; ^3^ Graduate School of Science and Engineering Yamagata University Yamagata Japan; ^4^ Toxicology Division Institute of Environmental Toxicology Joso Ibaraki Japan

**Keywords:** genetics, male fertility, reproduction

## Abstract

Subfertility and infertility are two major reproductive health problems in human and domestic animals. The contribution of the genotype to these conditions is poorly understood. To examine the genetic basis of male subfertility, we analyzed its relationship to sperm morphology in B10.MOL‐TEN1 mice, which shows high‐frequencies (about 50%) of morphologically abnormal sperm. Drastic histological changes were also found in the testis of the B10.MOL‐TEN1. Segregation analysis showed that the abnormal sperm phenotype in B10.MOL‐TEN1 was inherited and was predictably controlled by at least three loci. We also found that male fertility of this strain was normal. These findings indicate a complicated relationship between sperm morphology and male subfertility.

## Introduction

Reproduction is a highly regulated process that requires coordination of the functions of numerous genes. Infertility affects 10–15% of couples, and a male factor is estimated to be involved in nearly half of these cases (Visser and Repping 2010). Approximately 600 testis‐specific protein‐coding genes have been identified. Null mutations have been introduced in nearly 400 genes associated with spermatogenesis using knockout mouse technology (Matzuk and Lamb 2008; Jamsai and O'Bryan 2011; Massart et al. 2012). Information regarding genetic abnormalities in spermatogenesis derived using reverse genetics approaches is expected to help further understand male infertility. Recent advances in genetics have paved the way for the development of effective methods to study male infertility and subfertility. Accordingly, the number of “repro” mouse produced by JAX Reproductive Mutagenesis Program (Handel et al. 2006) reached more than a hundred, and a number of genes responsible for male infertility and subfertility have been identified (http://reprogenomics.jax.org). The bidirectional approaches described above are intended to study infertility caused by a single gene. However, the reproduction is temporally regulated by coordinated action of a number of genes.

Our approach was also genetic, but the phenotype was polygenic. Previously, 17 strains of mice were surveyed for frequency of abnormal sperm‐head morphology (Gotoh 2010) and found that B10.M and B10.MOL‐TEN1 (TEN1) strains were ranked first and second, respectively, with respect to the frequency of sperm‐head morphological abnormality. Analysis of B10.M strain revealed that the sperm phenotype was heritable. Further genetic analysis identified two causative loci, *Shm1* on Chromosome 1 and *Shm2* on Chromosome 4 (Gotoh et al. 2012). The B10.M strain showed low fecundity. The relationship between sperm‐head morphology and male subfertility is yet to be established. In this study, we analyzed the genetic control of sperm‐head morphological abnormality of TEN1 strain. The relationship between sperm‐head morphological abnormality and male fertility of B10.M and TEN1 strains was discussed.

## Materials and Methods

### Animals

All experiments were approved by the Institutional Animal Care and Use Committee of the National Institute of Agrobiological Sciences (study identification code #H18‐010). Animals were housed and cared for according to guidelines established by the Committee. B10.MOL‐TEN1 (TEN1) mice were from RIKEN BioResource Center (Tsukuba, Ibaraki, Japan). C3H/HeNCrlCrlj (C3H) mice were purchased from Charles River Japan (Yokohama, Japan). C57BL/10J (B10) mice were purchased from S. L. C. (Hamamatsu, Japan). B10.M/Sgn (B10.M) mice are maintained at our facility. Animals were maintained on a cycle of 12 h of light and 12 h of darkness under specific‐pathogen‐free conditions. A commercial mouse diet CRF‐1 (Charles River Japan, Yokohama, Japan) and water were provided.

### TEN1 strain

This strain was one of the congenic lines established by Dr Kazuo Moriwaki at the National Institute of Genetics, Japan (Mishima, Shizuoka, Japan) to define *H2* alleles on chromosome 17 in the Japanese wild mouse population (Shiroishi et al. 1982). The original donor was a wild non‐inbred animal (*Mus Musculus molossinus*). The wild‐derived *H2* allele was transmitted to B10 over 10 generations. Subsequently this strain has been maintained by sibling breeding.

### Sperm morphology test

**Figure 1:**
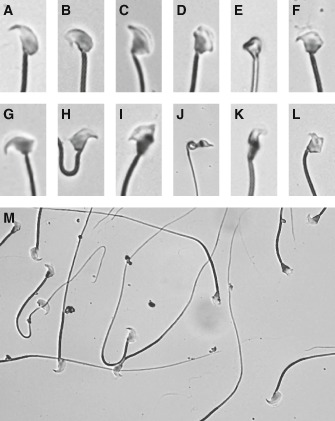
Sperm‐head morphology observed in the TEN1 strain. A Spermatozoon showing normal morphology. B–L Spermatozoa with abnormal sperm‐head morphology, including B a bent hook, C a shortened apical hook, D lack of the usual hook, E a round head; F–H ectopic attachment of the flagella and I–L amorphous heads. M, Mixture of spermatozoa showing various types of sperm‐head morphology.

Sperm samples were collected from 2‐ to 3‐month‐old male mice. The sperm‐head morphology test was performed as described earlier (Gotoh 2010; Gotoh et al. 2012). Briefly, to obtain sperm for the assessment of morphology, the epididymis was removed from one side and sperms were transferred into 1‐mL phosphate‐buffered saline (PBS, pH 7.0) solution containing 0.1% glucose (w/v). One microliter of the sperm suspension was spread on a glass slide, air‐dried, fixed using ethanol, and sperm morphology was assessed by a phase‐contrast microscopy (Optiphot‐2; Nikon, Tokyo, Japan) (400 × magnification). Two independent samples, each containing at least 200 sperm cells, were analyzed. The epididymis from the second side was used for sperm counting. The organ was transferred into a conical glass grinder and was manually homogenized. The total number of spermatozoa present in the epididymis was counted by using a Neubauer hemocytometer and a phase‐contrast microscope (400 × magnification). Classification of sperm‐head morphological abnormality (Fig. 1) was done as described earlier (Gotoh 2010; Gotoh et al. 2012).

### Animal mating for genetic analysis

The F1 hybrid males were generated by reciprocal crosses between C3H and TEN1 (CTF1; C3H female × TEN1 male, and TCF1; TEN1 female × C3H male). The F1 hybrids were then backcrossed either with C3H or with TEN1. The F2 hybrid males were generated by crosses between F1 females and F1 males.

### Preparation of genomic DNA

Mouse tails were resuspended in 300 μL Tris buffer (50 mM, pH 7.8) containing 100 mM ethylenediaminetetraacetic acid (EDTA), 100 mM NaCl, and 1% sodium dodecyl sulfate (SDS) (w/v). Proteinase K was then added to a final concentration of 500 μg/mL. The samples were shaken 1000 rpm, overnight at 56°C (Thermomixer comfort; Eppendorf, Hamburg, Germany). RNase A was then added to a final concentration of 10 μg/mL, and the samples were incubated for 1 h at 37°C. Following this, 300 μL DNA binding reagent containing 4.5 M guanidine hydrochloride, 0.5 M potassium acetate (pH 5.0), and 40 mg/mL Silica gel (Sigma‐Aldrich #288519, St. Louis, MO, USA) was added to the samples. The samples were then mixed by shaking for 1 min to allow DNA to bind to the silica gel. Silica gel particles were retrieved and washed three times with a solution containing 80% ethanol, 10 mM potassium acetate (pH 5.0), and 20 μM EDTA. After air‐drying, bound DNA was eluted from silica gel using the TE buffer (pH 8.0). DNA concentration was calculated indirectly from the absorbance at 260 nm measured using UV/Vis spectrophotometer (Gene Quant; Pharmacia Biotech, Cambridge, England).

### Linkage analysis

Genomic DNA was analyzed using microsatellite markers. Because the *H2* complex in mouse is located between the *H2‐K1* gene at 17.98 centimorgan (cM) (33996017‐34000333 bp; VEGA annotation of GRCm38) and the *H2‐T3* gene at 18.99 cM (36185572–36190287 bp), we chose polymorphic markers located around the *H2* complex. The specific markers used were D17Mit46 at 12.53 cM (25365940–25366175 bp), D17Mit22 at 17.98 cM (34333549–34333707 bp) and D17Mit139 at 27.40 cM (52659266–52659396 bp). Sequences of the primer pairs were as follows. fD17Mit, 5′‐TCCACCCCACTACCTGACTC‐3′ and 5′‐CCCTTCTGATGACCACAGGT‐3′; D17Mit22, 5′‐GGTAAGCATTAGATAGAGAG‐3′ and 5′‐TTATGATCTCCACACACGTG‐3′; D17Mit139, 5′‐AGACATGTGAGTACTGCACAGACA‐3′ and 5′‐ATGATGACATACCTCCTAGTAGTCCC‐3′. Primer pairs for microsatellite markers were purchased from Tsukuba Oligo (Ibaraki, Japan). Typically, a 10‐μL volume of each reaction mixture contained 10 ng of tail DNA, 50 mM KCl, 10 mM Tris‐HCl (pH 8.3), 2.5 mM MgCl_2_, 200 μM oligonucleotides, 200 μM dNTP, and 0.02 U AmpliTaq DNA polymerase (PerkinElmer, Norwalk, CT, USA). The cycling conditions were as follows: an initial step of denaturation for 2 min at 95°C; 45 s at 94°C (38 cycles); 1 min at 55°C; 1 min at 72°C; and a single final step of a 7‐min extension at 72°C. Polymerase chain reaction (PCR) products were separated by electrophoresis on a 4% MetaPhor agarose gel (FMC Bioproducts, Rockland, ME, USA) and visualized under UV light after staining with ethidium bromide.

### Male fertility analysis

Mature virgin C3H females aged between 8–12 weeks were used for the assay. A female mouse was mated with a male mouse. A vaginal plug was observed; 12 days later, the female mice were dissected, and the numbers of live embryos, placental remnants and implantation scars were counted according to the procedure used for the dominant lethal studies (Tezuka et al. 1978; Teramoto et al. 1980).

### Histology

Testes from TEN1 (*n* = 5) and B10 (*n* = 5) strains were fixed in Bouin's fluid, and 5 μm sections of paraffin‐embedded samples were stained with periodic acid‐schiff (PAS), and hematoxylin and eosin. Each seminiferous tubule was classified into one of the 12 stages of spermatogenesis as described previously (Russel et al. 1990). Differences in the histology of each stage between the mouse strains were compared. Testis histology was examined by light microscopy (Optiphot‐2; Nikon, Tokyo, Japan).

### Statistical analysis

Because both the B10.M and TEN1 strains are *H2* congenic strains on the background of the B10 genome, B10 was used as control. Data obtained in the present study were analyzed with routine multiple comparison tests as described earlier (Hojo et al. 2006; Aoyama et al. 2012) with slight modifications. Briefly, the data were first examined for equality of variance by the Bartlett's test (α = 0.05), which was followed by ANOVA when the group variance was homogeneous or by the Kruskal–Wallis test when the group variance was heterogeneous for analyzing differences among four strains or four crosses. When ANOVA was significant (α = 0.05), the Dunnett's test (parametric analysis) was conducted to detect differences between B10 and other strains of male mice or between C3H × B10 and other crosses α = 0.05). Differences between B10 and other strains or between C3H × B10 and other crosses were analyzed by the Dunnett‐type mean rank sum test (non‐parametric analysis, α = 0.05) if the Kruskal–Wallis test was significant (α = 0.05).

## Results

### Sperm‐head morphological abnormalities

The TEN1 strain displayed teratozoospermia. A wide variety of sperm‐head morphologies, including a shortened apical hook, the lack of the usual hook, a bent hook, a round head, a pin head, an amorphous head, and spermatozoa with ectopic attachment of the flagella were observed (Fig. 1). The observed variation in sperm‐head morphology was similar to that found in the B10.M strain (Gotoh 2010; Gotoh et al. 2012). In the TEN1 strain, frequencies of each abnormal type of sperm varies among individuals. The total frequency of various types of morphological sperm‐head abnormalities was found to be heritable. This feature was the same as that observed in the B10.M strain. In addition, besides sperm‐head abnormality, a considerable part of the sperm showed abnormality either in the midpiece or in tail. However, heritability of either midpiece‐abnormality or tail‐abnormality has not been established.

### Inheritance of the phenotype of sperm‐head abnormalities

**Figure 2:**
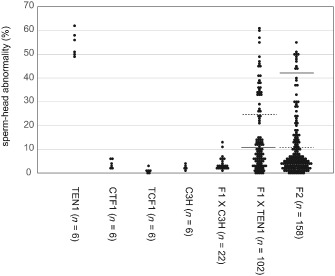
Segregation of abnormal sperm‐head phenotypes of the TEN1 strain induced by genetic crosses. Each dot indicates the percentage of abnormal sperm from an individual mouse. CTF1: C3H × TEN1; progeny from a cross between a C3H female and a TEN1 male. TCF1: TEN1 × C3H; progeny from a cross between a TEN1 female and a C3H male. F1 × C3H: progeny from a cross between an F1 (C3H × TEN1) female and a C3H male. F1 × TEN1: progeny from a cross between an F1 (C3H × TEN1) female and a TEN1 male. F2 (F1 × F1): progeny from a cross between an F1 male and an F1 female. A horizontal solid line in (F1 × TEN1) indicates that 50% of the number of animals is plotted above the line. This line was expected to divide animals with high or low frequencies of abnormal sperm‐head morphology, if the phenotype was controlled by a single recessive locus. Horizontal dotted lines indicate that 25% of the number of animals is plotted above the line. The dotted line in (F1 × TEN1) was expected to divide normal and abnormal animals, if the phenotype was controlled by two recessive loci. The dotted line in F2 was expected to divide normal and abnormal animals, if the phenotype was controlled by a single recessive locus. Horizontal solid double lines indicate that 6.3% of the number of animals is plotted above the line. The solid double line in F2 was expected to divide normal and abnormal animals, if the phenotype was controlled by two recessive loci.

Figure 2 shows the segregation of abnormal sperm‐head phenotypes of the TEN1 strain by genetic crosses between TEN1 and C3H strains. The mean frequencies of abnormal head morphology in the spermatozoa were 50.2% for TEN1 and 2.4% for C3H. The F1 hybrid males generated by reciprocal crosses (CTF1; C3H female × TEN1 male and TCF1; TEN1 female × C3H male) produced spermatozoa with a low frequency of abnormalities (average frequencies of 3.8% and 1.0%, respectively). We then backcrossed the F1 hybrids either with C3H or with TEN1. All of the male mice generated by backcrossing F1 hybrids with C3H produced sperm with a low frequency of abnormalities (mean, 4.1%). However, among the backcrosses of F1 hybrids to TEN1 mice, we observed segregation of the phenotypes of sperm‐head abnormalities to the high‐abnormality group and the low‐abnormality group. The frequency of males of the high‐abnormality group was 31%. In F2 hybrid mice, 18% of the males produced high levels of sperm with morphological abnormalities. The observed frequencies were found to be distributed continuously from the highest to the lowest.

### Sperm counts and reproductive organs

Epididymal sperm count, body weight, and reproductive organ weight were also analyzed (Table 1). No significant differences were observed in the epididymal sperm count, testis weight, seminal vesicle weight, or epididymis weight between the TEN1 and B10 males.

**Table 1:** Frequencies of abnormal spermatozoa, sperm count and weight of reproductive organs

Strain	Abnormal spermatozoa (%)	No. Spermatozoa (×10^6^/epididymis)	BW (g)	Testis (g)	SV (mg)	Epididymis (mg)
B10						
Mean	4.8	16.3	28.2	176	356	78.5
SD	0.4	2.2	1.5	16	55	6.5
*n*	6	6	6	6	6	6
TEN1						
Mean	50.2	17.5	34.6*	172	330	74.4
SD	3.7	3.3	2	12	75	4.1
*n*	6	6	6	6	6	6
B10.M						
Mean	61.5*	32.3*	33*	244	482*	95.5*
SD	2.4	1.7	1.4	6	52	10.7
*n*	6	6	6	6	6	6
C3H						
Mean	2.3	7.1*	31.8*	145	236*	56.2*
SD	0.5	2.3	1.4	27	35	4.4
*n*	6	6	6	6	6	6

Values represent mean, standard deviation (SD) and number examined.

Data were statistically analyzed by Dunnett's test following one‐way ANOVA or Dunnett‐type test following Kruskal–Wallis test.

Significantly different from control: *P ≤ 0.01.

### Chr 17 linkage

Because TEN1 was the *H2* congenic strain that showed a high frequency of sperm‐head abnormalities, we predicted that the gene responsible for the abnormal sperm‐head phenotype was located on Chr 17. However, analysis of this chromosome by using microsatellite markers showed no evidence of linkage of the abnormal sperm‐head phenotype to this chromosome.

### Male fertility

Male reproductive performances of the four strains, TEN1, B10.M, C3H and B10 were compared (Table 2), and no significant difference was observed in the number of ovulated oocytes, which were predicted by the number of corpora lutea, between the four groups. Significant loss of embryos was observed in all four groups in this study, on comparing the total number of corpora lutea and the total number of live embryos. The contribution of C3H females to this loss was not evident. On comparing both the mean number of live embryos and the rate of embryo survival between the four groups, B10.M males showed significantly low reproductive performance (*P* < 0.05). No significant difference in male fertility was observed between the C3H males, B10 males and TEN1 males.

**Table 2:** Comparison of male reproductive performance of four strains

Cross (female × male)	Mean no. embryos	Mean no. live embryos	Mean no. corpora lutea
C3H × B10			
Mean	8.1	7.6	10.3
SD	2.7	2.7	1.8
*n*	20	20	20
C3H × TEN1			
Mean	7.7	7.3	9.8
SD	2.7	2.7	1.7
*n*	20	20	20
C3H × B10.M			
Mean	3.8*	3.1*	10.8
SD	2.9	2.5	1.2
*n*	20	20	20
C3H × C3H			
Mean	8.4	7.6	10.6
SD	2.3	2.5	1.7
*n*	20	20	20

Values represent mean, SD and number examined.

Data were statistically analyzed by Dunnett's test following one‐way ANOVA or Dunnett‐type test following Kruskal–Wallis test.

Significantly different from control: *P ≤ 0.01.

### Histology of the testis

**Figure 3:**
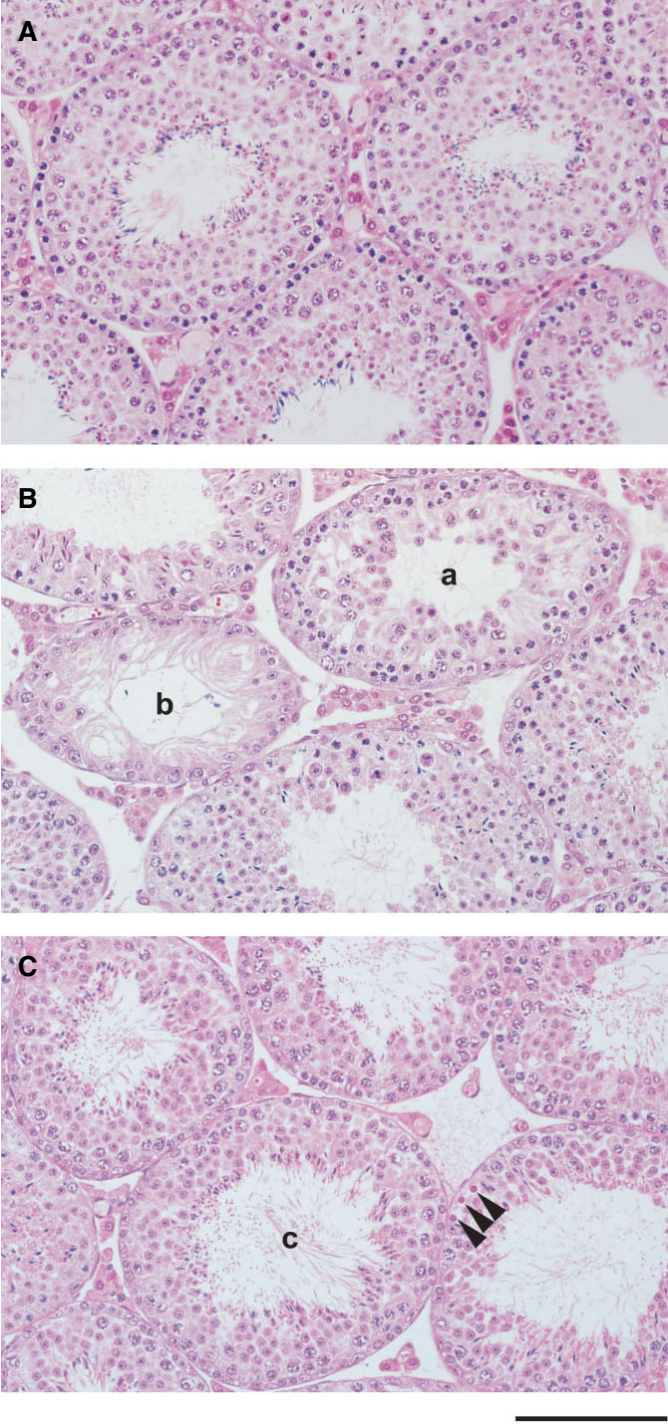
Testis histology. A, B10 testis. Normal histology. B, TEN1 testis. Various degenerative lesions of seminiferous epithelium were observed. (a) A seminiferous tubule incapable of determining the developmental stage of the spermatogenesis. Almost all of the young spermatocytes and elongated spermatids disappeared and large empty space was formed in the basal compartment of the seminiferous epithelium, while round spermatids were still observed. (b) A seminiferous tubule showing severe atrophy. This tubule lacks most germ cells and showed the feature called “Sertoli only tubule”. C, TEN1 testis including a normal seminiferous tubule. (c) A seminiferous tubule showing normal stage VII spermatogenesis. Spermatocytes in the adjacent tubule indicated by arrow‐heads have necrosed. Morphologically normal tubules were also found. Scale bar, 500 μm.

The histological observations of testis between the B10 and the TEN1 mice testes was compared (Fig. 3). Various degenerative lesions of seminiferous epitheliums including a single‐cell necrosis, an empty space, and a severe atrophy of a tubule were occasionally observed in the TEN1 testis (Fig. 3B). Morphologically normal epitheliums were also observed (Fig. 3C).

## Discussion

It is difficult to predict male reproductive fertility, even in experimental animals. Several types of sperm tests, including sperm morphology, sperm count, sperm mobility and others are used to assess male fertility (Aitken 2006; Lewis 2007). However, no reliable correlation between the results of sperm tests and observed fertility has so far been established (Guzick et al. 2001). Our study was intended to focus on genetic regulation of sperm morphology in mice as a model system to analyze the complicated reproductive regulation in mammals. In this study, we analyzed if the teratozoospermia of the TEN1 strain is heritable, and then predicted the number of loci regulating the sperm‐head abnormal phenotype. Male fertility tests were also performed.

Although reliable correlation between the results of sperm tests and observed fertility has not been established, sperm morphology, count, and motility are widely used to assess male fertility (Guzick et al. 2001). During our investigations on the relationship between sperm morphology and male fertility, we identified mutant strains showing high frequency of abnormal sperm morphology. Among these strains, B10.M and TEN1 were ranked first and second in the observed frequency of abnormal sperm morphology. Genetic analysis of B10.M revealed that the sperm phenotype was inherited (Gotoh 2010). Further quantitative trait loci (QTL) analysis and fine mapping identified that the sperm phenotype was controlled by two interactive recessive loci, *Shm1* on chromosome 1 and *Shm2* on chromosome 4 (Gotoh et al. 2012). The B10.M strain showed teratozoospermia, producing sperm cells displaying a wide variety of morphological abnormalities. Frequency of occurrence of each type of morphological abnormality varied among individuals. Collectively, our previous studies (Gotoh 2010; Gotoh et al. 2012) revealed that the total frequency of various types of sperm‐head morphological abnormalities was heritable. Severe male subfertility in the B10.M strain was evident. In the study reported here, we analyzed the TEN1 strain for sperm‐head morphological abnormalities and attempted to correlate the results with male fertility. The sperm phenotype of TEN1 was similar to that of B10.M. TEN1 strain displayed teratozoospermia. The types of sperm‐head abnormalities observed in TEN1 were identical to that observed in B10.M. The total frequency of the occurrence of abnormal sperm morphology was also found to be heritable. As shown in Figure 1, some sperm show abnormal shape not only in head but also in midpiece (Fig. 1 E, H, and J). Some connection between head abnormalities and midpiece abnormalities was suspected. However, heritability of midpiece abnormalities has not been detected. The cause(s) of midpiece abnormalities is apparently different from the loci causing sperm‐head abnormalities.

We tentatively set the threshold of high or low frequencies of sperm‐head abnormalities at and under 20%, respectively, for two reasons. First, because the maximum frequency of spermatozoa with sperm‐head abnormalities in individual mice within the F1 hybrid backcrossed with C3H group was below 20%. Second, because 98.9% of F2 animals produced from crosses between normal B6 and C3H strains showed sperm‐head abnormalities frequency of less than 20% (Gotoh and Aoyama 2012). The possibility of frequency of sperm‐head abnormalities of >20% without mutation was considered to be rare. As per these criteria, segregation of the sperm phenotype in TEN1 was different from that of B10.M. In the backcross of F1 offspring to TEN1, 50% of animals were expected to express an abnormal phenotype if one recessive locus was involved. If two recessive loci were involved, the segregation rate was expected to be 25%. In this cross, segregation of high and low frequencies of the phenotype was located between the 50% and 25% lines (Fig. 2). In the F2 animals, the predicted segregation rate for one recessive locus was 25%, and that for two recessive loci, 6.25%. If a 20% frequency of abnormality is tentatively assumed to be the threshold, it located in the middle of 25% and 6.3% lines (Fig. 2). The results indicated that neither the one recessive locus model nor the two recessive loci model explained the segregation of the phenotype of TEN1 strain. At least three loci are expected to be involved in this case. QTL analysis and genetic mapping are needed to identify the loci controlling sperm‐head abnormality.

The second difference was related to fertility. A complex relationship between sperm‐head abnormality and male fertility became apparent. The frequencies of sperm‐head abnormalities of B10.M and TEN1 were 61.5 ± 2.4% and 50.2 ± 3.7%, respectively (Table 1). Both scores were much higher than that for standard inbred strains including C3H (2.3 ± 0.5%) and B10 (4.8 ± 0.4%). B10.M males that showed high frequency of sperm head abnormality were diagnosed to be subfertile since they produced fewer live embryos (3.8 ± 2.9) on the 12th day after vaginal plug was observed when crossed with C3H females (Table 2). However, TEN1 males produced a normal number of live embryos (7.7 ± 2.7) irrespective of high frequency of sperm‐head abnormality observed in this strain. The significant difference in the embryo survival rates between B10.M males and TEN1 males could not be explained by 10% difference in sperm‐head abnormalities between the two strains. Qualitative difference between the spermatozoa of the two strains was not apparent from the observation by a light microscopy. Further studies are needed to elucidate the mechanisms underlying the differences in reproductive capacity of spermatozoa from the two strains.

The origin of loci causing sperm‐head abnormality phenotype of TEN1 is not known. Although TEN1 is an *H2* congenic strain on the B10 background, its sperm phenotype was not linked to *H2* complex on chromosome 17. Either introgression of chromosomal fragment(s) containing the mutation from the *H2* donor animal, or occurrence of *de novo* mutation(s) during/after establishment of congenic strain could be the origin of alleles responsible for the sperm phenotype.

A number of genes and loci important for male fertility have been identified (Handel et al. 2006; Matzuk and Lamb 2008). A single mutation in any one of over 400 genes or loci could result in male infertility or subfertility. The reproductive system is also regulated by a highly coordinated interaction of genes in a process known as epistasis. We found two interacting loci potentially responsible for the expression of subfertile phenotype of B10.M. Although not characterized, our findings suggest that at least three loci may be involved in determining the reproductive phenotype of TEN1. Our previous study examining sperm‐head morphology of F2 hybrid individuals generated by crossing two reproductively normal strains of mouse, C3H and C57BL/6J (B6), revealed that genetic factors other than mutations may contribute significantly toward determining the phenotype (Gotoh and Aoyama 2012). Reproductive function and sperm morphology of C3H and B6 strains were normal. However, approximately 20% of F2 animals showed higher levels of abnormalities than the parental strains, and approximately 1% of F2 animals showed very high (>20%) levels of abnormal sperm‐head morphology and histological changes in testis. Therefore inter genetic interaction of “normal” alleles from the two parental strains could affect sperm‐head morphology. In other words, epistasis of normal alleles of a set of responsible genes may have caused the abnormal phenotype of sperm without the involvement of mutations. The involvement of epistasis in reproductive performance in mouse has been reported (Peripato et al. 2004; Flachs et al. 2012). Our findings further confirm the notion that the reproductive system is controlled by coordinated action of numerous genes.

Histological observation found various degenerative lesions of seminiferous epithelium in the TEN1 testis. Normal epitheliums were also found. The degenerative changes were not observed through the entire tubule. In the seminiferous tubules, degree of degeneration varied widely from single cell necrosis to severe atrophy of the tubule. All necrotic cells observed were recognized clearly as germinal cells, and Sertoli cells were observed to be survived. A particular developmental stage of spermatogenesis has not been identified for the dead germ cells. Spaced observed in seminiferous epithelium were considered to be formed by the loss of syncytium of late spermatocytes and round spermatids. It seems that the degenerative changes take place occasionally or conditionally. In most of the testes of mice that were mutated, the genes specifically expressed in the spermatogenesis (Matzuk and Lamb 2008; Jamsai and O'Bryan 2011; Massart et al. 2012) showed histological changes in the entire testis, resulting in male infertility. Histological degeneration of the TEN1 testis is mosaic (Fig. 3), and TEN1 males were fully fertile (Table 2). From the observation of TEN1 testis, it was considered that the degeneration was brought about by the failure of communication between germ cells and Sertoli cells. Although no morphological changes were detected in Sertoli cells even in the severely atrophied seminiferous tubules, some functional abnormality of the Sertoli cell may play a key role in the degeneration in the testis of TEN1. Identification of responsible genes is required to understand the reproductive phenotype of the TEN1.

Sperm‐head abnormality in the B10.M has been shown to be controlled by two interacting loci, *Shm1* and *Shm2*. This strain also showed male subfertility. In contrast, the TEN1 strain was found to be fully fertile but showed high frequencies of sperm‐head morphological abnormality, predictably controlled by at least three loci. Identification of genes encoding loci controlling sperm‐head abnormality in both strains, and to investigate the function of the genes on male fertility will be needed to elucidate the issues of male reproduction.

## Disclosure

Authors have nothing to disclose and no conflicts of interest.
